# Comparison of Hemagglutination and Hemolytic Activity of Various Bacterial Clinical Isolates Against Different Human Blood Groups

**DOI:** 10.7759/cureus.489

**Published:** 2016-02-11

**Authors:** Rajkumar HRV, Ramakrishna Devaki, Venkataramana Kandi

**Affiliations:** 1 Department of Microbiology, Kamineni Academy of Medical Sciences & Research Centre; 2 Department of Biochemistry, Kamineni Academy of Medical Sciences & Research Centre; 3 Department of Microbiology, Prathima Institute of Medical Sciences

**Keywords:** haemagglutination, haemolysis, human blood group, red blood cells

## Abstract

Among the various pathogenic determinants shown by microorganisms hemagglutination and hemolysin production assume greater significance in terms of laboratory identification. This study evaluated the hemagglutination and hemolytic activity of various bacterial isolates against different blood groups. One hundred and fifty bacterial strains, isolated from clinical specimens like urine, pus, blood, and other body fluids were tested for their hemagglutinating and hemolytic activity against human A, B, AB, and O group red blood cells. Among the 150 isolates 81 were *Escherichia coli*, 18 were *Klebsiella pneumoniae*, 19 were *Pseudomonas aeruginosa*, 10 were *Pseudomonas *spp, six were *Proteus mirabilis,* and the rest 16 were *Staphylococcus aureus*. Nearly 85% of the isolates agglutinated A group cells followed by B and AB group (59.3% and 60.6% respectively). Least number of isolates agglutinated O group cells (38.0%). When the hemolytic activity was tested, out of these 150 isolates 79 (52.6%) hemolyzed A group cells, 61 (40.6%) hemolyzed AB group cells, 46 (30.6%) hemolyzed B group cells, and 57 (38.6%) isolates hemolyzed O group cells. Forty-six percent of the isolates exhibited both hemagglutinating and hemolytic property against A group cells, followed by B and AB group cells (28.6% and 21.3% respectively). Least number of isolates i.e., 32 (21.3%) showed both the properties against O group cells. The isolates showed wide variation in their hemagglutination and hemolytic properties against different combinations of human blood group cells. The study highlights the importance of selection of the type of cells especially when human RBCs are used for studying the hemagglutination and hemolytic activity of bacterial isolates because these two properties are considered as characteristic of pathogenic strains.

## Introduction

Hemagglutination and hemolysis of red blood cells (RBCs) are considered to be the characteristic features of some species of bacteria especially those belonging to the *Enterobacteriaceae *family. David C. Old in his review mentioned that bacteria with type I fimbriae agglutinate human cells strongly and sheep cells weakly [1]. Due to the presence of fimbriae many bacteria exhibit the property of hemagglutination and this has been used as an epidemiological marker along with other typing methods [2]. Several researchers studied the hemagglutinating property of various bacteria but most of them did not attempt to evaluate the red cell types especially when human cells were used for studies [3-4]. Few previous studies in the past have utilized and evaluated the hemagglutination and hemolytic activity of bacteria against O group cells and A group cells [5-6]. Another research in the past has used pooled red blood cells without actually revealing whether the pool contained single blood group cells or combined blood group cells [7]. Several previous reports are available in literature that have evaluated the hemolytic activity of various species of bacteria. These studies used both human as well as animal red cells with no specific mention of which human blood group was used [2, 8-9]. Only few reports are available in literature that have evaluated the hemagglutinating and hemolytic activity of various bacterial strains against different human red cell groups [3-9]. The present study attempts to evaluate hemolytic and hemagglutinating activity of bacterial clinical isolates against different human red blood cell groups including A, B, AB, and O.  

## Materials and methods

### Collection and preservation of blood cells

Four healthy volunteers belonging to the hospital staff were first clinically examined and laboratory parameters like hemoglobin, packed cell volume (PCV) and peripheral smear examination were carried out to rule out any red blood cell abnormality. Each volunteer belonged to one blood group. The study was approved by an institutional ethical committee (IEC) and an informed consent was obtained from the subjects included in the study. From each volunteer 10 ml blood was collected aseptically into a citrated tube to prevent coagulation. The specimens were centrifuged at 5000 rpm for five minutes to get a pack of red cells. The supernatant plasma was discarded and the RBCs were washed five times with phosphate-buffered saline (PBS) of pH 7.2. Later these cells were suspended in freshly prepared autoclaved Alsever’s solution (equal volume of 2.05% dextrose, 0.8% sodium citrate, 0.055% citric acid, and 0.42% sodium chloride). Whenever required, a small volume of RBCs stored in Alsever’s solution was taken into a test tube. After centrifugation, the RBCs were washed three times with PBS of pH 7.2, and then a working concentration of 3% V/V concentration was prepared for hemagglutination tests and 1% V/V concentration was prepared for hemolysis testing.

### Collection of bacterial isolates

Clinical specimens such as urine, blood, pus, and other body fluids received from different patients admitted under the various specialties of this hospital were processed using standard bacteriological techniques and the isolates were identified. These isolates were stored in semi solid nutrient agar for further studies.

### Procedure for hemagglutination tests

The isolates were subcultured in 5 ml of brain-heart infusion broth (Hi Media) and incubated overnight at 37^o^C. The culture was centrifuged to sediment the bacteria and the supernatant was discarded. The bacterial cells were suspended in PBS to a concentration of 5 X 10^10^ cells per ml (thick suspension). Chilled micro titre polystyrene plates were taken and 25 ml of chilled bacterial suspension and 25 ml of 3% concentration of chilled RBC from each group was added to separate wells and the trays were incubated at 4^o^C for one hour and the hemagglutination was observed as a complete even sheet of agglutinated RBCs. Positive tests were graded as 1+, 2+, 3+ or 4+ depending upon the clumping pattern when viewed on a mirror. The results were recorded for each isolate with different human blood group cells [10-11].

### Procedure for hemolytic activity of the cells

The same isolates, which were tested for hemagglutination with different human blood group cells, were also studied for hemolytic activity. The isolates were subcultured in 5 ml of brain-heart infusion (BHI) broth and incubated for 48 hours at 37^o^C. Two hundred micro litres of culture was distributed into each well of a microtire plate and an equal volume of 1% concentration of washed human RBCs of different blood groups were added and the plate was covered by a cellophane tape and incubated at 37^o^C for 24 hours and observed for hemolysis. If there was no haemolysis after 24 hours, the plate was kept in the cold room (2-6^o^C) for another 24 hours and observed for hemolyis. The controls consisted of 200 microlitres of sterile BHI broth with equal volume of washed human RBCs of different blood groups. The results were recorded for each isolate [10-11].

## Results

A total of 150 isolates were included in the study, which comprised *Eshcherichia coli* (81), *Klebsiella pneumoniae *(18), *Pseudomonas aeruginosa *(19), *Pseudomonas* spp (10), *Proteus mirabilis *(six) and *Staphylococcus aureus* (16).

The recovery of isolates from various specimens and the hemagglutination activity against different types of human RBCs is shown in Table 1.

**Table 1:** Specimen-wise incidence of hemagglutinating activity of bacterial isolates in relation to different blood group cells

Nature of the Specimens	Number of Isolates	Incidence of Hemagglutination Activity			
		A (n%)	B (n%)	AB (n%)	O (n%)
Urine	78	69 (88.4)	48 (61.5)	44 (56.4)	30 (38.4)
Pus	33	26 (78.7)	13 (39.3)	16 (48.4)	10 (30.3)
Blood	27	23 (85.1)	22 (81.4)	23 (85.1)	14 (51.8)
Others	12	10 (83.3)	06 (50.0)	08 (66.6)	03 (25.0)
Total	150	128 (85.3)	89 (59.3)	91 (60.6)	57 (38.0)

More than 50% of the isolates were from urine specimens and a majority of these strains showed hemagglutination with A group cells (88.4%) followed by B and AB group with an incidence of 61.5% and 56.4% respectively. The least number of urinary isolates showed hemagglutination with human O group cells (38.4%). A more or less same pattern was seen with isolates from other specimens like pus, blood etc., except that isolates from blood showed almost a same incidence of hemagglutination ranging from 81.4% to 85.1% against A, B, AB cells but only 51.8% isolates showed hemagglutination with human O cells. More number of isolates showed hemolytic activity against A group cells followed by AB group (40.6%), B group (30.6%), and O group (23.3%) in that order of frequency. A higher number of isolates from sputum and other body fluids (other than pus and blood) hemolyzed all groups of cells but again A group cells were hemolyzed predominantly by these isolates as shown in Table 2.

**Table 2:** Specimen-wise incidence of hemolytic activity of the bacterial isolates in relation to different human blood group cells

Nature of the Specimen	Number of Isolates	Hemolytic Activity			
		A (n%)	B (n%)	AB (n%)	O (n%)
Urine	78	39 (50.0)	26 (33.3)	29 (37.1)	17 (21.7)
Pus	33	16 (48.4)	07 (21.2)	13 (39.3)	09 (27.2)
Blood	27	15 (55.5)	08 (29.6)	12 (44.4)	04 (14.8)
Others	12	09 (75.0)	05 (41.6)	07 (58.8)	05 (41.6)
Total	150	79 (52.6)	46 (30.6)	61 (40.6)	35 (23.3)

The hemagglutinating activity of various bacterial species isolated is elaborated in Table 3.

**Table 3:** Hemagglutinating activity of clinical isolates in relation to different human blood group cells

Organism	Number of Isolates	Hemagglutinating Activity			
		A (n%)	B (n%)	AB (n%)	O (n%)
Escherichia coli	81	73 (90.1)	57 (70.3)	56 (69.1)	30 (58.0)
Klebsiella pneumoniae	18	10 (55.5)	02 (11.0)	05 (27.7)	04 (22.0)
Pseudomonas aeruginosa	19	19 (100.0)	16 (84.2)	17 (89.4)	10 (73.6)
Pseudomonas spp.	10	09 (90.0)	07 (70.0)	06 (60.0)	05 (50.0)
Proteus mirabilis	6	05 (83.0)	02 (33.3)	02 (33.3)	02 (33.3)
Staphylococcus aureus	16	12 (75.0)	05 (31.5)	05 (31.5)	05 (31.5)
Total	150	128 (85.3)	89 (59.3)	91 (60.6)	57 (38.0)

A majority of *Escherichia coli  *(90.1%) strains hemagglutinated A group cells followed by B (70.3%), AB (69.1%), and O (58.0%). A fewer number of *Klebsiella pneumoniae* isolates hemagglutinated all types of human RBCs. A significant number of *Pseudomonas aeruginosa* strains hemagglutinated all types of human RBCs. The least number of* Proteus mirabilis *strains hemagglutinated B, AB, and O group cells. More number of *Staphylococcus aureus* strains hemagglutinated A group cells but only 31% of isolates of *Staphylococcus aureus* exhibited hemagglutinating activity against B, AB, and O group cells.

The hemolytic activity of the isolates studied is shown in detail in Table 4.

**Table 4:** Hemolytic activity of the clinical isolates with different human blood group cells

Organism	Number of Isolates	Hemolytic Activity			
		A (n%)	B (n%)	AB (n%)	O (n%)
Escherichia coli	81	41 (50.6)	15 (18.5)	26 (32.0)	10 (12.3)
Klebsiella pneumoniae	18	03 (17.0)	01 (05.5)	02 (11.1)	02 (11.1)
Pseudomonas aeruginosa	19	18 (94.7)	16 (84.2)	17 (89.4)	12 (63.1)
Pseudomonas spp.	10	07 (70.0)	06 (60.0)	06 (60.0)	05 (50.0)
Proteus mirabilis	6	03 (50.0)	02 (33.3)	02 (33.3)	02 (33.3)
Staphylococcus aureus	16	07 (43.4)	06 (37.5)	08 (50.0)	04 (25.0)
Total	150	79 (52.6)	46 (30.6)	61 (40.6)	35 (23.3)

Similar to hemagglutination activity, A group cells were hemolyzed by 79 (52.6%) isolates, followed by the other groups. The least number of isolates showed hemolytic activity against O group cells. Among the species more number of *Pseudomonas aeruginosa *strains had least hemolytic activity against all types of human blood group cells as shown in Table 5.

**Table 5:** Incidence of bacterial strains having both hemagglutination and hemolytic activity against different human blood group cells

Organism	Total Number of Isolates	Hemagglutation and Hemolysis			
		A (n%)	B (n%)	AB (n%)	O (n%)
Escherichia coli	81	37 (45.6)	12 (14.8)	15 (18.5)	15 (18.5)
Klebsiella pneumoniae	18	01 (05.5)	-	-	-
Pseudomonas aeruginosa	19	18 (94.7)	16 (84.2)	15 (78.9)	09 (47.3)
Pseudomonas spp.	10	05 (50.0)	06 (60.0)	04 (40.0)	03 (30.0)
Proteus mirabilis	6	02 (33.3)	02 (33.3)	02 (33.3)	01 (16.6)
Staphylococcus aureus	16	06 (37.5)	07 (43.4)	05 (31.2)	04 (25.0)
Total	150	69 (46.0)	43 (28.6)	41 (27.3)	32 (21.3)

When a combination of various human blood group cells were tested for their hemagglutination and hemolytic activity by the same bacterial isolates a wide variation could be seen as shown in Table 6.

**Table 6:** Hemagglutinating activity of bacterial isolates with various combinations of human blood group cells

Blood Group Alone or in Combination	Number of Strains	Percentage of Hemagglutinating Strains
A	128	85.3
B	89	57.3
AB	91	60.6
O	57	38
A+AB	90	59
A+O	74	49
A+B	98	65
B+AB	71	47.3
B+O	68	45.3
AB+O	57	38
A+B+AB	23	15.3
A+B+O	19	12.6
A+O+AB	72	48
A+O+B+AB	63	42

The least number of strains hemagglutinated or hemolyzed when all blood group cells were combined and tested. The isolates could hemagglutinate or hemolyze the other combination of cells (two or three types combined) in the range of 30–40% as shown in Table 7.

**Table 7:** Hemolytic activity of bacterial isolates on various combinations of human blood group cells

Blood Group Alone or in Combination	Number of Strains	Percentage of Strains with Hemolytic Activity
A	79	52.6
B	46	30.6
AB	61	40.6
O	35	23.3
A+AB	57	38
A+O	63	42
A+B	38	25.3
B+AB	57	38
B+O	42	28
AB+O	45	30
A+B+AB	48	32
A+B+O	39	26
A+O+AB	28	18.6
A+B+O+AB	28	18.6

## Discussion

Hemagglutination and hemolysis of red blood cells indicate the pathogenic potential of bacterial species and that has been regularly evaluated. Bacteria agglutinate a number of different species of red blood cells such as that of human, rabbit, guinea pig, fowl, sheep, mouse, etc., and as many as 14 species of red blood cells have been tested for their hemagglutination. Hemagglutination of red blood cells by microbes was used as an epidemiological marker in conjunction with others. Only scanty literature is available regarding the use of human red cells for detecting hemagglutination and hemolytic activity, and the available studies failed to elaborate the specific group of RBCs used [3]. A previous research by Gupta, et al. in their study included 345 strains of *Escherichia* *coli* ​ and evaluated hemagglutination activity using human blood cells along with rabbit, sheep, and guinea pig RBCs. This study did not indicate the type of human RBCs used and found that 17.9% strains of *Escherichia* *coli* agglutinated human cells [3]. In our study only 38.0% of isolates could hemagglutinate human O blood group cells. Evans, et al. studied 611 strains of *Escherichia* coli isolated from urine and blood and noticed that 95% of the strains belonging to hemagglutination type VI hemagglutinated human A cells and 89% hemolyzed human A cells [12]. Barua, et al. also used human A cells along with guinea pig and monkey cells while studying eltor vibrio strains but they did not mention the incidence of hemagglutination with human A cells [4]. In their study Shrikhande, et al. evaluated the virulence factors in uropathogenic *Escherichia* *coli* and noted that 70% of their isolates hemagglutinated human O cells [13]. However, in the technique used by them there was no mention of testing the hemagglutination activity at 3-5^o ^C and further they did not use the centrifuged deposit of the bacterial cells to get a thick suspension as suggested by David C. Old [1]. 

Thus it is evident that there is a wide variation among the hemagglutination activity of bacterial strains against different types of human RBCs. Considering this variation we tested the same isolates against the four types of human red cells for hemagglutination and hemotytic activity. The study showed that 85.5% isolates hemagglutinated A group cells and 52.6% isolates hemolyzed them. The AB group cells were agglutinated by 60.6% isolates and hemolyzed by 40.6% isolates. Similarly 59.3% isolates agglutinated B group cells and 30.6% isolates hemolyzed them. Least number of isolates agglutinated or hemolyzed human O group cells (38.0% and 23.3% respectively). From our study results it can be assumed that most of the earlier authors might have used human O cells for testing the hemagglutination activity of the isolates because the incidence of hemagglutination with human cells in their studies was very low except the one study by Shirkhande, et al. which showed 70% hemagglutination with human blood O group cells and the discrepancy in their technique has been mentioned already [13].

It is therefore important to mention the source of human red cells when we perform hemagglutination or hemolytic studies with bacterial isolates as the hemagglutination or hemolytic activity of an isolate is influenced by the type of human cells used. Another observation that is very interesting in this study is that when the different blood group cells were combined and tested both hemaglutinating activity as well as hemolytic activity of the isolates was very low. Hypothetically this phenomenon might be due to the formation of fewer number of fimbriae which could not agglutinate and hemolyze all types of red cells resulting in invisible macroagglutination. Srikanth, et al. studied 144 strains of uropathogenic *Escherichia* *coli* using O and A group human cells along with cells of other species at a concentration of 4% RBCs. This study failed to evaluate the incidence of hemagglutination with O and A cells. They observed that 72/144 strains were showing hemolytic activity [14].

From the available literature it is evident that most of the previous studies reported the hemolytic activity of gram negative bacilli using sheep blood cells which detects alpha-hemolysin [15-16]. Johnson, et al. studied both environmental as well as clinical isolates and found that all strains of *Vibrio* *vulnificus* were hemolytic against pooled human RBCs of unknown type [6]. In our study we detected all four types of hemolysins (alpha, beta, gamma, and delta) using individual human red cells and observed that a majority of the isolates were hemolytic to human A group cells with an incidence of 52.6% followed by other cell types. We observed that by and large the human cell types are less hemolytic. Similar observations were made by Nischal, et al. who studied the hemolytic activity of *Enterococci* using different human red cells. They observed that A and O cells were hemolyzed by more number of strains and that a 0.5% cell concentration gave a higher incidence of hemolytic strains than other concentrations i.e., 1.0%, 1.5%, and 2.0% cells [9]. In our study only A group cells were hemolyzed by more number of isolates. This might be due to the amount and type of hemolysin produced by the isolate. We could not find any related article available in literature to compare our data.

## Conclusions

In conclusion the present results indicate that most of the isolates included in our study were hemagglutinating and hemolyzing human A group cells. The combination of different human blood cell types showed a wide variation in hemagglutination and hemolytic activity of the same isolates that were tested against individual human red cell types. Further studies in this regard are warranted to understand the clinical relevance of these observations.
